# Albuminuric Retinitis of Pregnancy

**Published:** 1883-11

**Authors:** 


### Abstracts.

ALBUMINURIC RETINITIS OF PREGNANCY.—DR. RYERSON, of Toronto, writes:

"Mrs. E., aged 22, was referred to me by Dr. Temple on June 1, 1881, with the statement that the urine contained a large amount of albumen. The patient stated that her sight had been failing for about a month. She said she could see the sides of an object, but not the center, and complained of flashes of light in the dark. She had frontal headache, sometimes severely. She had no pain in the eyes. There was a great deal of nausea and vomiting. She was in the fourth month of her first pregnancy. With the right eye she saw fingers at five feet, and read 16 Jäger; with the left she saw fingers at three feet, and read 20 Jäeger. With the ophthalmoscope I observed in the right eye a well marked stellate arrangement of deposits about the yellow spot, with numerous patches scattered about the retina. The optic disc was somewhat swollen and indistinct in its outline. The appearances in the left eye were very similar, with the addition of numerous small hæmorrhages in the lower half of the fundus.

Dr. Temple informs me that shortly after this she was seized with convulsions, and had a miscarriage. She made a good recovery, and when I saw her again on August 4 the swelling of the optic disc had greatly diminished, the scattered patches were less marked, but stellate patches in the region of the macula were about the same as when first seen. In the right eye two veins apparently contained thrombi. The vision was, with the right eye, $\frac{20}{50}$, 16 Jäeger; with the left eye, $\frac{20}{50}$, 16 Jäeger. She could manage to write a letter. From Dr. Temple I learn that she regained good vision, but did not myself see her again. In a few months the unfortunate woman became pregnant again, although warned of the danger; convulsions supervened, and in one of them she died.

Remarks.—It would be of considerable interest to learn in what proportion, and in what class of cases of albuminuri of pregnancy, retinitis occurs. That it does not necessarily occur, I know, having attended some years ago two cases in which there was no complaint of trouble of the vision. One case, a woman of about thirty years, in her fourth pregnancy, made a good recovery. The other had uræmic convulsions, and died. I did not use the ophthalmoscope, but relied upon the patient's statements, the cases having occurred in my pre-ophthalmoscopic days."—*British Medical Journal.*

